# Adult age-differences in subjective impression of emotional faces are reflected in emotion-related attention and memory tasks

**DOI:** 10.3389/fpsyg.2014.00423

**Published:** 2014-05-14

**Authors:** Joakim Svärd, Håkan Fischer, Daniel Lundqvist

**Affiliations:** ^1^Aging Research Center, Karolinska Institutet and Stockholm UniversityStockholm, Sweden; ^2^Department of Psychology, Stockholm UniversityStockholm, Sweden; ^3^Department of Clinical Neuroscience, Karolinska InstitutetStockholm, Sweden

**Keywords:** emotion, faces, arousal, aging, subjective rating, attention, categorical perception, memory

## Abstract

Although younger and older adults appear to attend to and remember emotional faces differently, less is known about age-related differences in the subjective emotional impression (arousal, potency, and valence) of emotional faces and how these differences, in turn, are reflected in age differences in various emotional tasks. In the current study, we used the same facial emotional stimuli (angry and happy faces) in four tasks: emotional rating, attention, categorical perception, and visual short-term memory (VSTM). The aim of this study was to investigate effects of age on the subjective emotional impression of angry and happy faces and to examine whether any age differences were mirrored in measures of emotional behavior (attention, categorical perception, and memory). In addition, regression analyses were used to further study impression-behavior associations. Forty younger adults (range 20–30 years) and thirty-nine older adults (range 65–75 years) participated in the experiment. The emotional rating task showed that older adults perceived less arousal, potency, and valence than younger adults and that the difference was more pronounced for angry than happy faces. Similarly, the results of the attention and memory tasks demonstrated interaction effects between emotion and age, and age differences on these measures were larger for angry than for happy faces. Regression analyses confirmed that in both age groups, higher potency ratings predicted both visual search and VSTM efficiency. Future studies should consider the possibility that age differences in the subjective emotional impression of facial emotional stimuli may explain age differences in attention to and memory of such stimuli.

## Introduction

The effects of age on the processing of emotional facial expression have been investigated in a broad range of emotion-cognition domains, such as attention (e.g., Isaacowitz et al., 2006), memory (e.g., Mather and Carstensen, 2003), and categorical perception (Kiffel et al., 2005). However, age differences in the subjective emotional experience of facial expressions have been less extensively investigated. For example, studies of age differences in ratings of valence (pleasantness/unpleasantness), arousal (active/passive), and potency (weak/strong) (see e.g., Osgood, 1966; Russell, 2003) of such stimuli are rare. Consequently, little is known about whether age-related differences in the subjective emotional impression—that is, the valence, arousal, and potency rating—of emotional stimuli is associated with emotional behavior (e.g., memory, attention).

### Age and emotional processing of facial expression

Studies on the processing of emotional facial expression, including studies on recognition, attention, and memory, have revealed that aging is associated with less efficient processing of negative than positive faces. More specifically, older adults have repeatedly demonstrated poorer recognition than younger adults of expressions that are fearful (e.g., McDowell et al., 1994; Calder et al., 2003; Keightley et al., 2006; Horning et al., 2012; Svärd et al., 2012; Suzuki and Akiyama, 2013), angry (McDowell et al., 1994; Calder et al., 2003; Ebner and Johnson, 2009; Suzuki and Akiyama, 2013), and sad (McDowell et al., 1994; Calder et al., 2003; Keightley et al., 2006; Horning et al., 2012; Suzuki and Akiyama, 2013). However, they have a preserved or even increased ability to recognize facial expressions that are happy (e.g., McDowell et al., 1994; Calder et al., 2003; Svärd et al., 2012, but see also Horning et al., 2012; Suzuki and Akiyama, 2013) and disgusted (Calder et al., 2003; Horning et al., 2012; Suzuki and Akiyama, 2013). This pattern of results from individual studies has been confirmed in a meta-analysis by Ruffman et al. (2008), who found that the largest age-related decrease occurred in the recognition of angry, fearful, and sad faces and that less of a decrease occurred in the recognition of happy and surprised faces.

A similar age-by-emotion interaction has been demonstrated in memory for emotional faces. For instance, Mather and Carstensen (2003) have demonstrated that although older adults remembered more positive than negative faces, there was no such difference among younger adults. Similarly, Ebner and Johnson (2009) have reported that older adults'; memory for angry faces was poorer than their memory for happy and neutral faces. Enhanced memory for negative faces has also been reported among younger but not older adults (Grady et al., 2007; but see also D'Argembeau and van der Linden, 2004; Fischer et al., 2010). Studies of the effects of age on VSTM for emotional material have shown either a general decrease with age (Borg et al., 2011) or age-by-emotion interactions, findings that are consistent with the above-mentioned pattern of effects. For example, Mikels et al. (2005) found that younger adults were able to maintain intensity judgments of negative emotional pictures to a larger extent than of positive emotional pictures, but the opposite was true for older adults. A similar finding was reported in a study by Langeslag and van Strien (2009) in which younger adults exhibited enhanced VSTM performance for unpleasant but not pleasant stimuli, and older adults exhibited similar VSTM for both types of stimuli. However, to the best of our knowledge, no studies have investigated the effects of age on VSTM of emotional faces.

The attention literature shows that older adults preferentially focus on happy rather than sad faces, whereas younger adults do not (Isaacowitz et al., 2006). Moreover, older adults focus away from negative faces in negative-neutral face-pairs, but younger adults do not (Mather and Carstensen, 2003). However, when the task requires detection of negative (angry) schematic faces, these positivity preferences in older adults diminish (Hahn et al., 2006; Mather and Knight, 2006; Ruffman et al., 2009; Lundqvist et al., 2013a).

The findings from recognition, attention, and memory studies all show that the processing of negative but not positive facial expressions decreases with increasing age. This age-by-emotion interaction is often discussed in the theoretical framework of the *socio-emotional selectivity theory* (SST; Carstensen et al., 1999), which proposes motivational shifts away from negative and toward positive stimuli in the processing of emotional material as a function of limited time perspectives (see e.g., Carstensen, 2006). However, the contribution of subjective emotional impression to emotional processing remains relatively unexplored in the literature on age and emotion. This is intriguing because, as described in the next section, a growing body of research on younger adults shows that subjective emotional impression affect emotional processing.

### Effects of arousal on emotional processing

Several reviews of studies on the attention-emotion domain in younger adults show that a stimulus'; score on emotional arousal measures is more important for processing efficiency than the valence of the stimulus (in particular, see Lundqvist et al., 2013b; also c.f. Phelps and LeDoux, 2005; Lang and Bradley, 2010; Mather and Sutherland, 2011; Harmon-Jones et al., 2012). Recent results support the idea that emotional arousal is also important in older adults; they suggest that arousal may be the main emotional impression factor involved in age-related flattening of affect (Lundqvist et al., 2013a). More specifically, these results showed a significant age-related reduction in minimum and maximum ratings on emotional arousal and potency measures in response to facial stimuli. The results also showed a relationship between arousal ratings, potency ratings, and attention measures for angry (but not for happy) faces, such that higher ratings on those scales were associated with shorter reaction times (RTs) in a visual search task. A growing body of research on younger adults also points to emotional arousal as an important factor in VSTM performance. More specifically, both maintenance (Langeslag et al., 2009; Lindström and Bohlin, 2011) and immediate recall and recognition performance seem to be boosted by emotionally arousing content (Jackson et al., 2009; Langeslag et al., 2009).

A potential mechanisms through which arousal may impact task performance in the reported tasks is by means of activation in core affective arousal-related brain regions such as the amygdala, PAG and orbitofrontal cortex, which, in turn, influence activity in cortical regions and visual cortex to facilitate both higher cognitive processing and more basic visual perception (see e.g., Phelps and LeDoux, 2005).

In summary, studies in younger adults show an association between emotional arousal and efficiency in processing emotional information in both the attention and memory domains. With the exception of one study that found that the association between arousal and attention is preserved in older age (Lundqvist et al., 2013a), the effect of arousal on emotional processing in other emotion-cognition domains remains largely unexplored in the literature on aging.

### A three dimension approach to the study of emotion

In the cognition-emotion literature, the dimension of valence has historically been the emotional dimension of largest interest (see e.g., Lundqvist et al., 2013b), whereas the dimensions of arousal and potency have been investigated to a much lesser degree. All three dimensions reoccur in the literature (since the mid-fifties) as the three main underlying dimensions of affective/emotional responses to a wide array of emotional stimuli (e.g., Osgood and Suci, 1955; Lundqvist et al., 1999). The steady reoccurrence demonstrates the importance of these dimensions in socio-emotional interactions, and makes it meaningful and motivated to include all three dimensions in studies of emotion-cognition relationships.

### Implications for the study of effects of age on emotional processing

The findings of an effect of emotional impression on behavior point to the importance of stimulus selection in studies of emotional behavior in younger and older adults. If emotional faces convey different impression to younger and older adults, this may in turn affect attention and memory processing. Given that both memory literature (which indicates that participants remember material and events of emotional character better than those that are neutral; see e.g., Buchanan, 2007, for a review) and recent attention literature, which has found that arousal affects attention processing (e.g., Lundqvist et al., 2013b), a decrease in emotional impression might thus dampen the effect of emotion on memory performance and attention processing. By assuming that younger and older adults perceive emotional material equally in terms of the key dimensions of emotional impression, researchers risk misattributing age effects in emotional behavior.

### Extension to another emotion-cognition domain

Categorical perception is another emotion-cognition domain that remains relatively unexplored, both in terms of age and arousal effects. The phenomenon of categorical perception refers to the occurrence of perceived categories along a linear continuum of physically changing stimulus (see e.g., Bornstein and Korda, 1984 for a more detailed description). Such phenomena have been shown in studies of color perception (e.g., Bornstein and Korda, 1984), speech perception (Liberman et al., 1957), and facial expression recognition (Etcoff and Magee, 1992). Although the physical differences between pairs of emotional faces in the Etcoff and Magee study (1992) were held constant, discrete perceived boundaries occurred for a variety of the emotion-emotion and emotion-neutral face pairs. Even though this finding of categorical perception of emotional facial expressions has been replicated in numerous studies of younger adults (e.g., Calder et al., 1996; Young et al., 1997; Campanella et al., 2002), less is known about how this type of categorical perception might vary as a function of age. To the best of our knowledge, only one study has investigated the effects of age on categorical perception of emotional facial expressions. That study showed that although categorical perception of identity was affected by age, categorical perception of emotional expressions seemed to remain intact (Kiffel et al., 2005).

### The aim and hypothesis of the study

The overall aim of this study was to extend earlier studies on subjective emotional impression and attention in older people. The study had three specific aims. The first was to investigate differences between older and younger adults in subjective emotional impressions. The second was to explore whether any such differences were mirrored in the emotional-cognitive tasks of attention, categorical perception, and VSTM. The third was to analyze whether any differences in the impression were associated with differences in task performance.

The literature on emotion indicates that the emotional properties of stimuli influence stimulus processing in many different domains, from perception to visual attention and memory (see e.g., Phelps and LeDoux, 2005). In this study, these different domains were represented by our categorical perception task (perception), visual search task (attention), and VSTM task (memory). Because of our previous results (Lundqvist et al., 2013a) and the above-mentioned reviews, we expected to replicate our earlier findings, which showed lower ratings in emotional impression of facial expression in older adults and an age-independent association between arousal ratings and visual search performance. We further expected to find performance differences across the perception, attention, and VSTM tasks, in keeping with the idea that high arousal scores are associated with high performance on tasks, and vice versa. This predicted decline in categorical perception would contrast with the Kiffel et al. (2005) finding that categorical perception of emotional expressions seems to remain intact with age. However, their study did not include angry faces, and it is angry faces that we predict will garner lower emotional ratings in older but not younger adults. Because of our previous results (Lundqvist et al., 2013a) and the above-mentioned reviews, we expect arousal to be the dimension that most strongly predicts task performance. However, because of our previous findings (Lundqvist et al., 2013a) and suggestions that at least a third emotional dimension should be incorporated in the study of emotions (Russell and Mehrabian, 1977; Fontaine et al., 2007), we also expected emotional potency to be involved in task performance.

Specifically, we expected: (1) to find lower ratings (i.e., arousal, potency) of emotional stimuli in older than younger adults, (2) to demonstrate that such lower ratings (i.e., arousal, potency) are associated with response latencies on the visual search task, and (3) that subjective emotional impression of facial expressions would be associated with performance on VSTM and categorical perception tasks. We did not have any predictions whether these associations are restricted only to angry faces, because previous results have showed that high arousal ratings of both angry and happy faces visual are associated with increased search efficiency (Lundqvist et al., 2013b).

## Methods

### Participants

Table 1 shows the characteristics of the participants in this study. Although younger adults scored higher than older adults on the anxiety items on the Hospital Anxiety and Depression Scale (HADS), this difference most likely did not affect behavioral results because no age-related differences in either trait anxiety (STAI-T) or state anxiety (STAI-S) were measured at test. All participants had normal or corrected-to-normal vision. Written informed consent was collected prior the tests and participants received two movie vouchers for their participation. The same participants were enrolled in all four tasks. The study was approved by the local ethics committee.

**Table 1:** **Participant characteristics by age group**.

	**Younger**	**Older**	***p*-value**	***d***
	**M**	**M**		
Age in years (*SD*)	25.2 (10.5)	70.5 (2.8)	<0.001	13.127
Sex	21 F, 19 M	24 F, 15 M		
Education in years (*SD*)	14.6 (2.9)	14.5 (2.9)	0.831	0.049
MMSE (*SD*; range)	29 (0.9; 26–30)	28.7 (1.4; 24–30)	0.295	0.237
HADS^a^ (*SD*; range)	5 (3.5; 0–13)	3.4 (3.0; 0–15)	0.028	0.509
HADS^d^ (*SD*; range)	3.1 (2.3; 0–12)	2.9 (2.2; 0–10)	0.653	0.102
STAI-T (*SD*)	47.7 (4.8; 27–59)	47 (3.1; 42–56)	0.390	0.196
STAI-S (*SD*)	30.1 (5.8; 20–42)	29 (5.2; 20–40)	0.381	0.201

MMSE, mini mental state examination (Folstein et al., 1975); HADS, hospital anxiety and depression scale (Zigmond and Snaith, 1983); STAI, state-trait anxiety inventory (Spielberger et al., 1988); -T, Trait; -S, State;

a anxiety items;

d depression items; F, female; M, male; ^*^p < 0.05.

### Materials

#### Rating and visual search tasks

**Figure 1:**
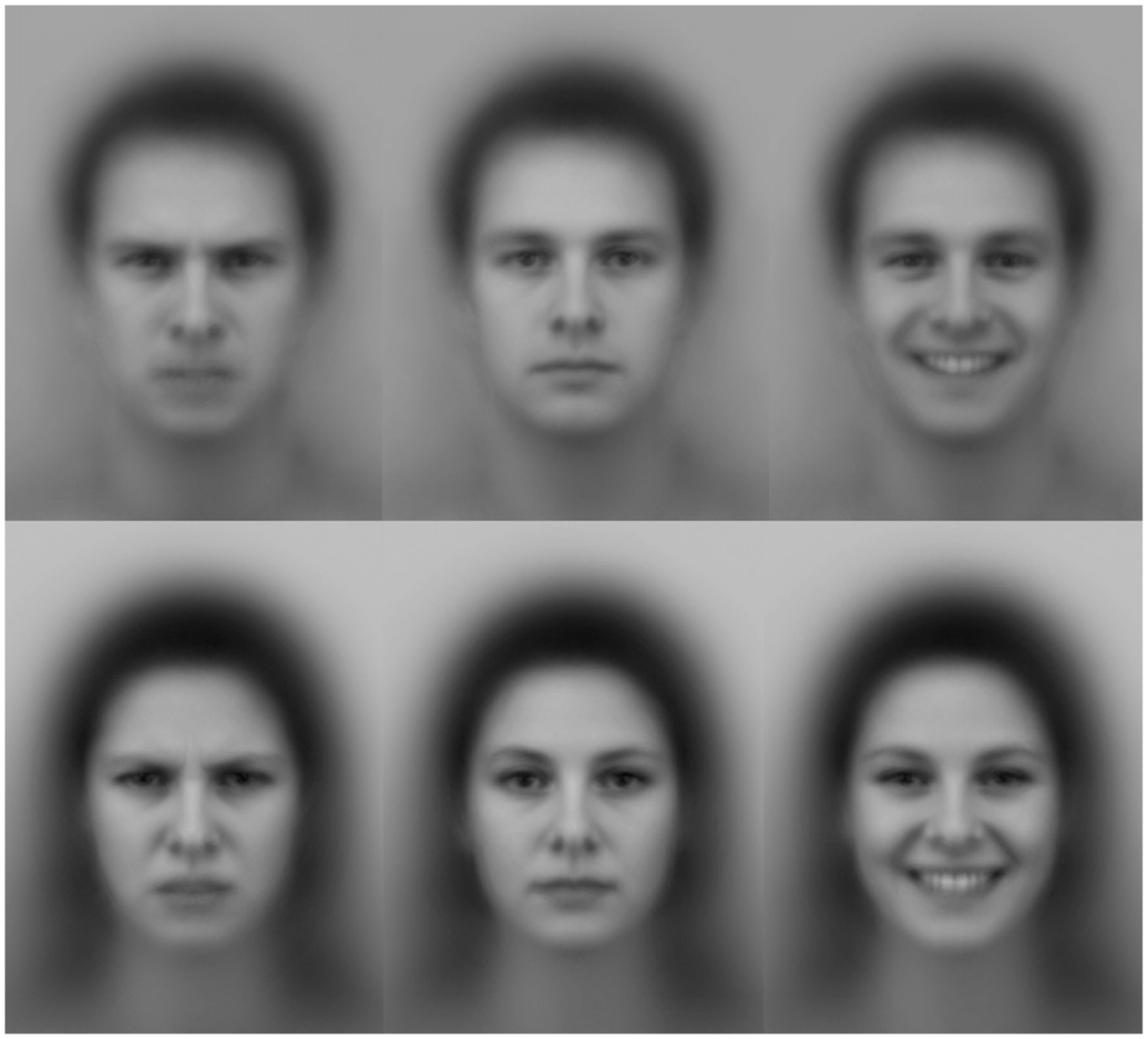
**Examples of the face stimuli used in all four tasks**. Note: Faces from the Averaged Karolinska Directed Emotional Faces set (Lundqvist and Litton, 1998).

Faces from the Averaged Karolinska Directed Emotional Faces set (AKDEF; Lundqvist and Litton, 1998) were used in the study. The AKDEF faces consist of an averaged female and an averaged male face, each composed of 35 individuals (see Figure 1). In the emotional rating task and in the visual search task, angry, happy, and neutral expressions from both the averaged female face and the averaged male face were used as stimuli. Ratings of and visual search performance for fear, disgust, sadness, and surprise were also collected but not analyzed in the current study.

#### Categorical perception and visual short-term memory tasks

**Figure 2:**
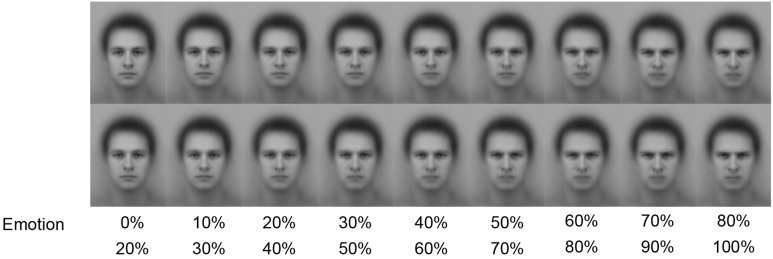
**Examples of face pairs used in the categorical perception task (presented vertically)**. At study, the faces in each pair were presented side by side.

The stimuli shown in Figure 1 were also the basis of the stimuli in the categorical perception and VSTM tasks. However, to enable investigation of categorical boundaries and VSTM performance on a continuum from a neutral to an emotional facial expression, the faces were modified to show emotional expressions at various intensities between 0 and 100%. The modification of the faces was performed using SqirlsMorph 2.1 (http://www.xiberpix.net/), and key points were used to guide the morph between facial features (e.g., lips, mouth shape, eyes, nose wrinkles, facial outline). For the categorical perception task, 9 face pairs differing 20% in intensity (i.e., 20 steps apart along the 100-step morph) were created in the neutral-to-angry and neutral-to-happy continua for the female and male averaged faces respectively (see Figure 2). The size of each face was 7.5 × 10.5 cm.

### Procedure

To minimize the risk of initiating any potential biases caused by elaborative processing, the rating task was performed last in the sequence. Although the order in which the tasks were conducted was visual search, categorical perception, visual short-term memory, and rating, to facilitate reading and interpretation of the manuscript, the rating experiment is presented first. The order of the tasks was the same for all participants. At study, participants sat on a chair in front of a computer screen (HP Compaq LA2405wg) at a distance of approximately 0.5 m. Behavioral responses were collected by key presses on an external keyboard. The experiment was programmed using Macromedia Director MX 2004 software (Macromedia Inc.). Total duration of the experiment was approximately 75 min. The visual search task took 30 min, and the rating, VSTM, and categorical perception tasks each took 15 min. Short breaks were given between the tasks to prevent fatigue.

#### Emotional rating task

During the rating task, facial stimuli (Figure 1) with full emotional expressions (100% intensity) were presented one at a time on the computer screen. Participants used a visual analog scale (VAS) to rate the three key emotional dimensions arousal (active-inactive), valence (pleasant-unpleasant), and potency (weak-strong) of seven expressions on male faces and seven on female faces (a total of 42 faces). Cursors on three separated horizontal lines were used for the ratings. The scales ranged from −1 to 1 (actual values were not visible to the participants).

The three VAS scales were presented below the face and maneuvered with the right and left arrow keys. Participants were informed that the task was self-paced. However, they were encouraged to base their rating on the first impression of a face and not spend exaggerated time thinking. Once ratings on the three scales were completed, participants pressed the spacebar to continue to the next trial. The order and polarity of scales were randomized between trials.

#### Visual search task

During the visual search task, circular displays of 6 faces were presented to the participants. In half of the trials (so-called target-absent trials), all faces in the array had the same emotional expression, while in the other half (so-called target-present trials), one of the faces was different from the other (e.g., an angry face among neutral faces).

Participants were instructed to determine whether each array consisted of faces that were all the same (in which case they were to press a button marked “all same” with their left index finger), or whether one of the faces was different from the others (in which case they were to press a button marked “one deviant” with their right index finger). They were instructed to respond as quickly and accurately as possible. A fixation cross initiated each trial. During target-absent conditions, all faces were afraid, angry, disgusted, happy, neutral, sad, or surprised. During target-present conditions, the background would either be *neutral*, with afraid, angry, disgusted, happy, sad, or surprised faces as the deviating target, or *emotional* (afraid, angry, disgusted, happy, sad, or surprised faces), with neutral as the deviating target. The target face was presented once at each position in the array. The presentation of trials was blocked by condition (emotional background or neutral background) and sex of faces (female or male). Thus, four blocks that included a total 288 faces (2 sexes, 6 expressions, 6 positions, 4 blocks) were used. As soon as a key was pressed (“all similar” or “one deviant”), the trial was terminated and the next trial was initiated. This article only includes information about the analyses of angry and happy emotional faces to make the effects of emotional stimuli (angry, happy) congruent with the stimuli used in the other tasks in the study. The order of blocks was randomized for each participant.

#### Categorical perception task

During the categorical perception task, pairs of faces were presented side by side on the screen. The physical difference between the faces in each pair was always 20%. Thus, a face showing 0% emotionality (i.e., a neutral face) was always paired with a face showing 20% emotionality, a face 10% emotionality was always paired with a face showing 30% emotionality, and so on (see Figure 2). Each pair was shown twice in a randomized order. Presentation of stimuli was however blocked for emotional expression and for the sex of the depicted face. Thus, each of the four blocked conditions (male, neutral to angry; male neural to happy; female, neutral to angry; and female, neutral to happy) contained 2^*^9 pairs, or a total of 72 pair of faces. The order of blocks was balanced over participants.

Participants were instructed to decide whether the two faces in each pair showed the same facial expression or not. Participants gave their answers by pressing keys labeled “same” or “different.” Thus, a shift in Categorical perception from neutral to an emotional expression was investigated. Because pilot tests before start of the study showed that some participants tended to respond “different” on trials where the intensity rather than expression differed, it was emphasized that the task was to judge the facial expression of the face and not whether the expression showed the same intensity or not. The faces stayed on the screen until participants gave their response, which terminated the trial and initiated the next trial.

#### Visual short-term memory task

In the VSTM task, an emotional face (angry or happy) that varied in expressed emotional intensity in 10% intervals ranging from 10 to 90% was displayed on the screen for three seconds before it disappeared. After a one second interval, a face again appeared on the screen, but at a different (random) intensity level. Participants were instructed to remember the first face and upon display of the second (random) face, adjust the face';s intensity level until it matched that of the first face. Intensity adjustments were made with the right and left arrow keys. After completing the adjustments, participants pressed the enter key, and a new trial was initiated. Each intensity interval was shown twice in a randomized order. The presentations were however blocked for emotional expression and for the sex of the depicted face. Thus, each of the four blocked conditions (male, neutral to angry; male neural to happy; female, neutral to angry; and female, neutral to happy) contained 2^*^9 faces, or a total of 72 presented faces. The order of blocks was balanced over participants.

### Data preparation and statistical analysis

#### Rating task

Average ratings for the female and male faces were calculated separately for the emotional dimensions valence, arousal, and potency before statistical analyses were run.

#### Visual search task

Separate ANOVAs were run for reaction times (RTs) and accuracy. Correct responses were included in the analysis of RTs. Before analysis, raw RT data was log10-transformed to achieve normal distribution of data. Furthermore, individual outliers (*M* >< ±3 * SD) were replaced by (*M* ± 3 * SD). However, to facilitate interpretation of the results, raw values are presented in the text and figures.

#### Categorical perception task

To analyze categorical perception data, a peak intensity interval (e.g., 30–50) was identified for each participant (separately for each of the two emotions). Within each emotion, data for the two AKDEF stimuli genders were collapsed before identifying the peak. Since there were clear differences in how many responses of “different” a participant tended to give (across intervals), the peak was identified by first identifying the interval with the most responses of “different” for that participant and that emotion (e.g., neutral to angry). If there were several responses with the same frequency across a span of intervals (e.g., 20–40; 30–50; 40–60) an average interval was calculated (here 30–50). The most frequent reported “different” pair was thus assumed to be the peak at which categorical perception occurred (Beale and Keil, 1995). The category border itself was assumed to be in the middle of the peak interval (e.g., at 40% if the peak was identified in a 30–50% pair). Thus, peaks were identified for angry and happy faces separately for each individual participant.

#### Visual short-term memory task

To investigate VSTM performance, the intensity level of the face during encoding was subtracted from the intensity levels reported by the participant. That is, if the face showed 20% intensity at encoding and the participants adjusted the face to show 28% intensity at retrieval, the error score was 8%. Further, to transform all error scores to a positive value, all answers were square root transformed [√ (answer*answer)] at the level of individual responses. The transformation was made after initial analyses where it was discovered that un-transformed errors made in both directions (e.g., −20 and +20% for two trials within one emotion) led to the false impression that this condition was reported with perfect accuracy (*M* = 0%). Thus, to study effects of age and emotional impression on VSTM performance, transformed data were used in analyses.

## Results

To investigate age effects on specific emotion-cognition domains separated ANOVAs were planned for the rating, visual search, VSTM, and categorical perception tasks. Regression analyses were carried out on each of the three behavior tasks to study the effects of emotional impression on emotional behavior. Because of the exploratory nature of this first study on emotional impression and its relation to different emotional behaviors, we decided that the risk of making a type I error was less important than the risk missing important findings. Therefore, corrections for multiple comparisons were not performed.

For all analyses, regular degrees of freedom are reported together with observed significance levels after Greenhouse-Geisser correction. Results were considered significant at *p* ≤ 0.05.

### ANOVAs

A multivariate analysis revealed a significant effect of age on the combined task performance, *F*_(4, 63)_ = 24.62, *p* < 0.001, η^2^ = 0.610. Analysis of each tasks confirmed the effect of age on rating, *F*_(1, 66)_ = 13.22, *p* = 0.001, η^2^ = 0.167, visual search, *F*_(1, 66)_ = 65.60, *p* < 0.001, η^2^ = 0.498, categorical perception, *F*_(1, 66) = 12.92_, *p* = 0.001, η^2^ = 0.164, and VSTM, *F*_(1, 66)_ = 23.15, *p* < 0.001, η^2^ = 0.260, performance. Separated repeated ANOVAs were carried out to study effects of age and emotion on each of the tasks.

#### Rating task

**Figure 3:**
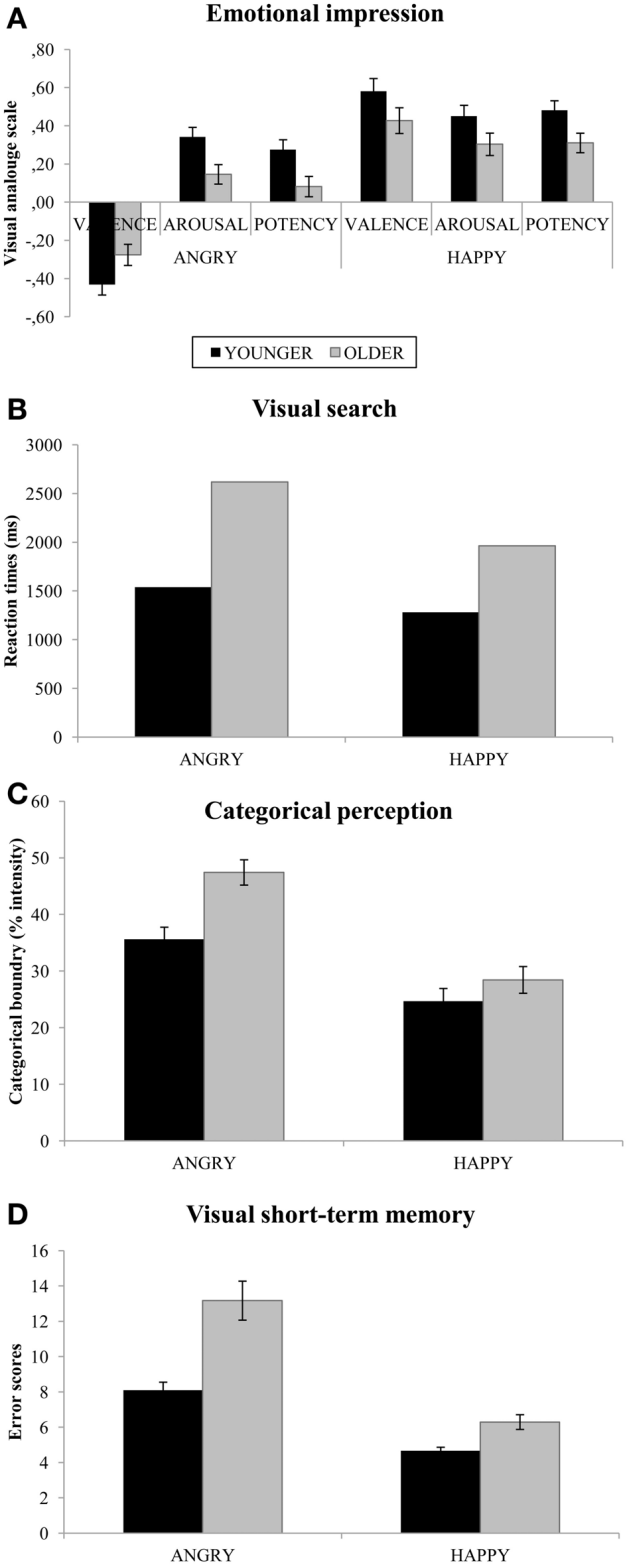
**Mean (SE) performance in the rating task (A), visual search task (B), categorical perception task, (C) and visual short-term memory task (D) separately for expression and age**. Note. Error bars represent standard error. Results of the ANOVAs and *t*-tests for age differences are presented in the result section.

Separate ANOVAs were carried out for the emotional dimensions of valence, arousal, and potency. In the ANOVAs, age (young, old) was used as the between-subject variable and emotion (angry, happy) was used as within-subject variables. Planned *t*-tests were conducted to study the effect of age on each dimension of emotion. These tests were conducted separately for angry and happy faces. The results are presented in Figure 3A.

***Valence.*** The main effect of Emotion *F*_(1, 77)_ = 133.73, *p* < 0.001, η^2^ = 0.635, showed overall higher ratings for Happy (*M* = 0.51, *SD* = 0.43) compared to Angry (*M* = −0.35, *SD* = 0.35) faces. There was also an interaction effect between Age and Emotion *F*_(1, 77)_ = 4.35, *p* = 0.04, η^2^ = 0.053, that showed that the largest decrease in responsiveness among Older adults was for Angry faces. Follow up *t*-tests for the interaction revealed that Older adults ratings (*M* = −0.28, *SD* = 0.38) were lower than those of Younger adults (*M* = −0.31, *SD* = 0.31) for Angry faces, *t*_(77)_ = 1.99, *p* = 0.05, *d* = 0.087. For happy faces, there was no difference, *p* = 0.11.

***Arousal.*** The ANOVA on arousal revealed a main effect of Emotion, *F*_(1, 77)_ = 7.80, *p* = 0.007, η^2^ = 0.092, that showed higher ratings for Happy (*M* = 0.38, *SD* = 0.37) compared to Angry (*M* = 0.25, *SD* = 0.33) faces. The main effect of Age, *F*_(1, 77)_ = 7.96, *p* = 0.006, η^2^ = 0.094, showed that Older adults (*M* = 0.22, *SD* = 0.23) gave lower ratings than Younger adults (*M* = 0.40, *SD* = 0.30). The planned *t*-tests revealed that Older adults ratings (*M* = 0.15, *SD* = 0.27) were lower than those of Younger adults (*M* = 0.34, *SD* = 0.36) for Angry faces, *t*_(1, 77)_ = 2.74, *p* = 0.008, *d* = 0.597. For Happy faces, Older adults tended to give lower ratings (*M* = 0.30, *SD* = 0.38) than Younger adults (*M* = 0.45, *SD* = 0.35), which resulted in a trend toward an age difference, *t*_(1, 77)_ = 1.78, *p* = 0.078, *d* = 0.411.

***Potency.*** The main effect of Emotion, *F*_(1, 77)_ = 16.95, *p* < 0.001, η^2^ = 0.180, showed higher ratings for Happy (*M* = 0.40, *SD* = 0.33) than for Angry (*M* = 0.18, *SD* = 0.34) faces. The effect of Age, *F*_(1, 77)_ = 12.91, *p* = 001, η^2^ = 0.144, showed that Older adults (*M* = 0.20, *SD* = 0.19) gave lower ratings than Younger adults (*M* = 0.38, *SD* = 0.26). The planned *t*-tests revealed that Older adults ratings (*M* = 0.08, *SD* = 0.29) were lower than those of Younger adults (*M* = 0.27, *SD* = 0.38) for Angry faces, *t*_(1, 77)_ = 2.6, *p* = 0.011, *d* = 0.533. For Happy faces, Older adults'; ratings (*M* = 0.31, *SD* = 0.33) were lower than those of Younger adults, (*M* = 0.48, *SD* = 0.31), *t*_(1, 77)_ = 2.38, *p* = 0.020, *d* = 0.531.

#### Visual search task

***Accuracy (%).*** The ANOVA on Accuracy revealed a main effect of Emotion, *F*_(1, 77)_ = 13.84, *p* < 0.001, η^2^ = 0.152, showing that across age Happy faces (*M* = 96.10, *SD* = 0.07) were detected with higher accuracy than Angry faces (*M* = 90.71, *SD* = 0.14). There was also a main effect of Age, *F*_(1, 77)_ = 8.32, *p* = 0.005, η^2^ = 0.098, showing that across emotions, Older adults (*M* = 96.20, *SD* = 0.01) detected a deviant emotional face among neutral distractors more accurately than did Younger adults (*M* = 90.70, *SD* = 0.10).

***Reaction time (ms).*** The ANOVA on RTs revealed a main effect of Emotion, *F*_(1, 77)_ = 118.23, *p* < 0.001, η^2^ = 0.606, showing that Happy faces (*M* = 1618, *SD* = 525) were detected faster than Angry faces (*M* = 2071, *SD* = 925). A main effect of Age, *F*_(1, 77)_ = 76.30, *p* < 0.001, η^2^ = 0.498, showed that Older adults (*M* = 2290, *SD* = 702) were slower than Younger adults (*M* = 1411, *SD* = 338) in detecting a deviant face. There was also an interaction between Age and Emotion, *F*_(1, 77)_ = 6.51, *p* = 0.013, η^2^ = 0.078, showing that although Older adults were slower than Younger adults in detecting both Angry and Happy faces, this effect was more pronounced for Angry faces (Figure 3B).

#### Categorical perception task

Because of equipment failure, data from two older women were not collected. In addition, seven (3 younger and 4 older adults) non-responders (i.e., participants who did not report any different response at all) were excluded from the analysis (11.4% excluded in total). Thus, 37 (20 female) younger and 34 (20 female) older adults were included in this analysis.

The ANOVA on Categorical perception data demonstrated a main effect of Emotion, *F*_(1, 69)_ = 44.72, *p* < 0.001, η^2^ = 0.393, showing that the shift in Categorical perception from neutral to an emotional expression occurred earlier for Happy (*M* = 26.5, *SD* = 13.8), than for Angry (*M* = 41.3, *SD* = 14.2) faces. The main effect of Age, *F*_(1, 69)_ = 11.88, *p* < 0.001, η^2^ = 0.147, revealed that across emotions (Angry and Happy), Older adults categorical shift from a neutral to an emotional face occurred at a later stage (*M* = 37.9, *SD* = 10.8) along the continuum compared to Younger adults (*M* = 30.2, *SD* = 8.2). (Figure 3C). In addition, there was also an trend toward an Age by Emotion interaction, *F*_(1, 69)_ = 3.22, *p* = 0.077, η^2^ = 0.045.

#### Visual short-term memory task

Data inspection revealed two outliers (whose mean score was <> ± 3 SD), which were excluded from analyses together with one participant with missing data (3.8% excluded in total). Thus, analyses were computed for 40 younger (21 female) and 35 older (21 female) adults. The statistical design of the ANOVA on VSTM was a 2 (Emotion: Angry, Happy) by 9 (Intensity levels: 10–90%) design with Age (Young, Old) as a between-group variable.

The results revealed a main effect of Emotion, *F*_(1, 73) = 104.23_, *p* < 0.001, η^2^ = 0.588. Across intensity levels, both Younger and Older adults performed better (fewer errors) for Happy (*M* = 5.32, *SD* = 1.90) than for Angry (*M* = 10.18, *SD* = 4.80) faces. There was also a main effect of Age, *F*_(1, 73) = 26.83_, *p* < 0.001, η^2^ = 0.269: Older adults (*M* = 9.32, *SD* = 3.13) made more errors than Younger adults (*M* = 6.38, *SD* = 1.67). In addition, there was an interaction between Age and Emotion, *F*_(1, 73) = 9.99_, *p* = 0.002, η^2^ = 0.120: although Older adults performed worse than Younger adults for both emotions, the poorer performance of Older adults was more pronounced for Angry faces (Figure 3D). There was also a main effect of Intensity, *F*_(8, 584) = 2.33_, *p* = 0.029, η^2^ = 0.031. Contrast tests showed that in both age groups and for both emotions, participants made significantly more errors at the 90% intensity level than at any other level, *ps* < 0.05.

### Regression analyses

A multivariate multiple regression analysis revealed an association between emotional impression (all dimensions) and the combined task performance, *F*_(3, 64) = 3.10_, *p* = 0.033, η^2^ = 0.127. Analysis of each tasks confirmed the association between emotional impression and visual search, *F*_(1, 66) = 6.92_, *p* = 0.011, η^2^ = 0.095, VSTM, *F*_(1, 66)_ = 6.79, *p* = 0.011, η^2^ = 0.093, but not categorical perception, *F*_(1, 66) = 0.85_, *p* = 0.361, η^2^ = 0.013, performance. Separated regressions were carried out to study associations between specific dimensions and tasks.

Regression analyses were conducted for each of the behavior tasks (for angry and happy faces separately). In these analyses, three models were used to explore the relationship between emotional impression and behavior. Model one was univariate; each emotional rating scale was tested alone as the predictor. Model 2 was multivariate; all three scales were included as predictors. Thus, in Model 2, any effects were adjusted for the influences of the other scales. Finally, in Model 3, age was included as a predictor, which means that any effect in this model holds even after controlling for age effects. Models 2 and 3 were performed using the enter method.

**Figure 4:**
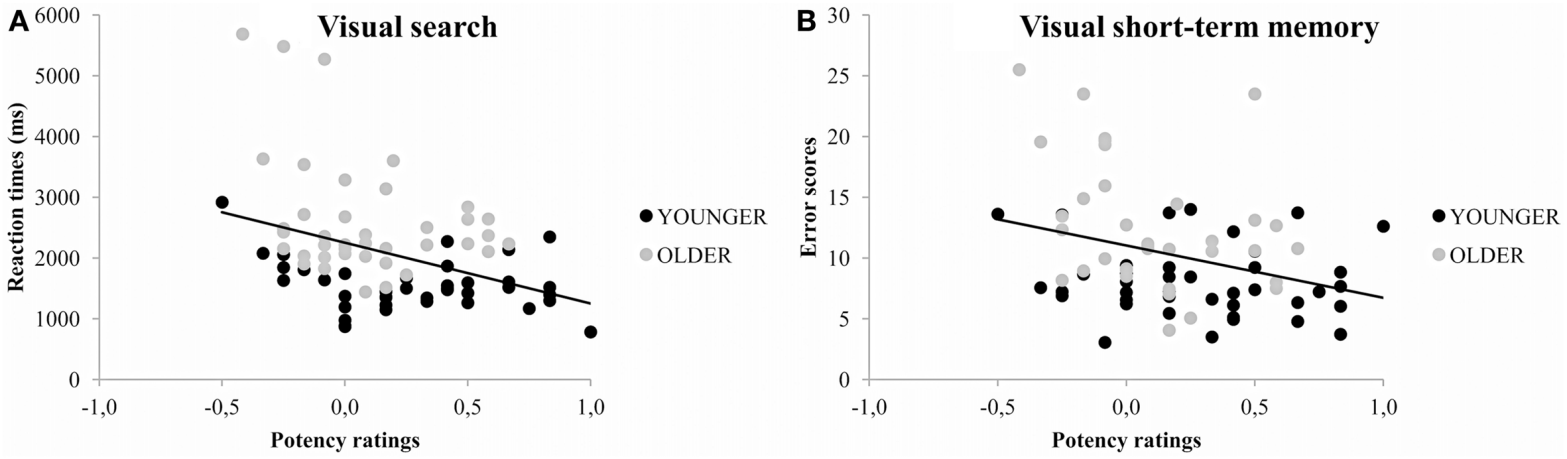
**Scatterplots for potency ratings and emotional behavior tasks, separately for visual search (A) and visual short-term memory (B) performance for angry faces across age**. Note. Visual search: *r* = −0.372; Visual short-term memory: *r* = −0.312.

The univariate analyses for Angry faces revealed that both Arousal and Potency ratings were associated with Visual search performance (*ps* < 0.05). In addition, Potency ratings were also associated with VSTM performance (*p* < 0.05). The association between Arousal ratings and emotional behavior vanished when the analyses were adjusted for the influences of the other scales (Model 2) and the influences of the other scales and age (Model 3). However, the association between Potency ratings and Visual search and VSTM performance remained significant even after these adjustments (*ps* < 0.05). These age-independent relationships can be seen in Figure 4 in the different location of younger (middle to top left area) and older adults'; (middle to bottom right area) scores along the regression line. For Happy faces, there were no associations between any of the emotional rating scales and emotional behavior tasks (*ps* > 0.05). Table 2 gives information regarding the predictor variables used in the different models.

**Table 2:** **β-coefficients (SE) and bivariate correlations for rating scores associated with emotional behavior**.

**Predictors**	**Angry**			**Happy**		
**By task**	**Model 1^a^**	**Model 2^a^**	**Model 3^a^**	**Model 1^a^**	**Model 2^a^**	**Model 3^a^**
**VISUAL SEARCH (RT)**						
Arousal	−0.25 (0.06)^*^	−0.08 (0.06)	0.03 (0.05)	−0.14 (0.04)	−0.15 (0.05)	−0.09 (0.04)
Potency	−0.36 (0.05)^**^	−0.31 (0.06)^*^	−0.21 (0.05)^*^	−0.11 (0.05)	−0.08 (0.05)	0.07 (0.04)
Valence	0.13 (0.05)	0.01 (0.05)	−0.07 (0.04)	−0.03 (0.04)	0.08 (0.04)	0.11 (0.03)
**VISUAL SHORT-TERM MEMORY (ERROR SCORES)**						
Arousal	−0.09 (1.68)	0.12 (1.97)	0.21 (1.78)	−0.10 (0.61)	−0.07 (0.73)	−0.04 (0.69)
Potency	−0.31 (1.53)^*^	−0.37 (1.86)^*^	−0.32 (1.67)^*^	−0.19 (0.66)	−0.20 (0.74)	−0.11 (0.72)
Valence	0.11 (1.57)	0.04 (1.62)	−0.04 (1.47)	−0.03 (0.52)	0.09 (0.62)	0.12 (0.59)
**CATEGORICAL PERCEPTION (PEAK)**						
Arousal	−0.19 (5.05)	−0.17 (6.26)	−0.12 (5.82)	0.10 (4.41)	0.05 (5.14)	0.07 (5.13)
Potency	−0.12 (4.97)	−0.03 (6.11)	0.07 (5.78)	0.04 (5.07)	−0.03 (5.66)	0.00 (5.67)
Valence	0.07 (5.03)	0.00 (5.43)	−0.03 (5.05)	0.15 (4.04)	0.14 (4.84)	0.16 (4.82)
**Correlations**	**Angry**			**Happy**		
**Between predictors**	**Arousal**	**Potency**	**Valence**	**Arousal**	**Potency**	**Valence**
Arousal	–	0.539^**^	−0.306^*^	–	0.379^**^	0.522^**^
Potency		–	−0.288^*^		–	0.410^**^

a Model 1 was univariate; Model 2 was multivariate, adjusted for the other rating scales; and Model 3 was additionally adjusted for age.

** p ≤ 0.01;

* 0.01 < p ≤ 0.05.

## Discussion

The emotional rating task revealed that older adults rated facial expressions as less emotional than younger adults, and these age differences were more pronounced for angry than happy faces. This age-by-emotion interaction was also evident in visual search and visual short-term memory performance. Regression analyses confirmed that in both age groups, higher potency ratings were the foremost predictor of both visual search and VSTM efficiency for angry faces but not for happy ones. We will briefly discuss the results separately for each task as well as results regarding the associations between subjective emotional impressions and emotional behavior.

### Specific emotion-cognition domains

#### Rating

The results of the rating task revealed a weaker emotional impression of facial expressions in older than in younger adults, which was more pronounced for angry than happy faces (Figure 3A, left panel). Older adults rated the angry faces as significantly less unpleasant (valence), less active (arousal), and weaker (potency) than did younger adults. A similar pattern was found for happy faces, but only the difference in the potency measure was statistically significant (see Figure 3A, right panel). These results are in line with previous results from our group (Lundqvist et al., 2013a), which show that the emotional impression of faces diminishes with advancing age, and that this dampening is most pronounced for angry faces.

#### Visual search

An age-by-emotion interaction in RTs revealed that although older adults were generally slower than younger adults, this effect was more pronounced for the detection of angry faces. To the best of our knowledge, this article is the first to report an age-by-emotion interaction in visual search efficiency for emotional faces. It should be noted that our results (which used photographic stimuli) are in disagreement with the results of overall age and emotion effects from previous studies that used schematic (Hahn et al., 2006; Mather and Knight, 2006; Ruffman et al., 2009; Lundqvist et al., 2013a) and photographic (Ruffman et al., 2009) faces. Our finding of faster detection of happy than angry faces is also in disagreement with the findings of the above-mentioned studies. The finding of an angry superiority effect (ASE) in previous studies and a happy superiority effect (HSE; for an overview, see Lundqvist et al., 2013a). in the present study is linked to the emotional impression of the stimuli used and will be discussed below.

The pattern of results in the visual search tasks shows that the older participants generate markedly longer RTs. However, it also shows that this slowness is not without advantages, as the older group also responds with greater accuracy. Whether the high accuracy is a side-effect from the slow responses, or part of a strategy is difficult to say from the present data, since we do not assess or address response strategies.

#### Categorical perception

The results of the categorical perception task show that both angry and happy faces were perceived categorically by both age groups. These results are in line with the results of several other studies on younger adults (e.g., Calder et al., 1996; Young et al., 1997; Campanella et al., 2002) and on older adults (Kiffel et al., 2005). The present study adds new information to the existing literature by showing that the perceptual category boundaries can vary as a function of both age and emotion. The current study used a neutral expression as a reference and paired it separately with an angry or a happy expression; the results showed reliable differences in the locus of boundaries for angry and happy faces. More specifically, our results showed that in both age groups, the locus of the boundary was further forward for angry than for happy faces, indicating that the angry faces were processed more ambiguously than the happy ones. In light of the results from the other tasks, the trend toward an interaction between age and emotion in this task is noteworthy. Although it did not reach a conventional statistical threshold, the relationship agrees with the general pattern of results in this article, which shows that older adults process angry faces less readily than younger adults.

#### Visual short-term memory

The results of the VSTM task showed that the older adults performed less accurately than younger adults, and the difference in accuracy was greater for angry than for happy faces. Earlier studies have shown that older adults have a decreased ability to maintain unpleasant non-facial stimuli (Langeslag and van Strien, 2009) and negative intensity judgments (Mikels et al., 2005). Our results extend these previous findings into the domain of emotional expression by showing a relatively larger reduction in VSTM performance for angry than for happy facial expressions. The results agree on the general pattern of results in this article, which shows that older adults process angry faces less readily than younger adults.

### Emotional impression-behavior relationships

A central aim of this study was to replicate and extend our previous findings (Lundqvist et al., 2013a) of an association between emotional impression and visual search efficiency to include new cognitive-emotion domains. As seen in Table 2, the emotional dimension of potency was associated with the results of two out of three emotional behavior tasks for angry faces.

The association between arousal and potency ratings and visual search efficiency replicates our previous findings of a similar relationship for schematic emotional faces (Lundqvist et al., 2013a). In that study, higher arousal and potency ratings were correlated with lower search times for angry but not happy faces. In accordance with the findings of the current study, this association was age-independent, which indicates that visual search performance in both younger and older adults is associated with potency measures. In the present analyses, this relationship can also be seen in Figure 4A in the differing location of younger (middle to top left area) and older adults'; (middle to bottom right area) scores along the regression line. Both our previous findings (Lundqvist et al., 2013a) and our present results indicate that although older adults generally have lower visual search efficiency than younger adults, search efficiency is associated with the same underlying emotional dimensions (potency and arousal) regardless of age. This notion is also partially supported by the work of Leclerc and Kensinger (2008, 2010), who found that when arousal levels of positive and negative stimuli were equated, all valence effects were absent. Importantly, the lack of interactions with age demonstrated an age-independent reliance on arousal in visual search performance.

Within the visual search literature, researchers usually report on whether their results show an ASE or a HSE (see Visual Search. above). In those terms, the present results demonstrate an HSE. A recent review article by Lundqvist et al. (2013b) concluded that when participants compared two facial expressions, the expression with the highest arousal ratings was detected most efficiently, regardless of whether that expression was happy or angry. The Lundqvist et al. (2013b) article may thus explain the disagreement between our findings of an HSE and previous studies'; findings of an ASE. In accordance with the notion that the facial expression with the highest arousal rating is detected most efficiently, the current study showed a HSE in both arousal ratings and visual search performance. Thus, the current results help generalize the role played by arousal in visual attention to the later part of the adult life-span. In terms of behavior and related brain processes, the HSE effect is potentially associated with a more attention driven positivity bias associated with activity in the dorsolateral prefrontal cortex (Lindquist et al., 2012), a more elaborative cognitive bias associated with activity in the ventromedial prefrontal cortex (Ebner et al., 2012) and/or a more perceptually driven bias associated with activation in the occipital cortex (Lindquist et al., 2012). A recent review by Lundqvist et al. (2013b) however shows that both the HSE concept and its rivaling concept ASE may be confound by an uncontrolled influence from the arousal factor. The present data (showing overall lower scores on arousal for angry compared to happy faces indicates that the present HSE finding may well be explained by the arousal differences between the angry and happy stimuli.

Like attention processing, VSTM performance was associated with the potency ratings of stimuli, such that higher potency ratings were associated with fewer errors. This age-independent association can be seen in Figure 4B in the different location of younger (middle to top left area) and older adults'; (middle to bottom right area) scores along the regression line. Like the emotion-attention relationship, this relationship indicate that although older adults generally have lower VSTM efficiency than younger adults, VSTM efficiency is associated with the same underlying emotional dimensions (potency and arousal) regardless of age. These findings of age-independent associations might contribute to the understanding of the preserved processing of the happy facial expression that is reported in recognition (e.g., Ruffman et al., 2008), attention (e.g., Isaacowitz et al., 2006), and memory (e.g., Mather and Carstensen, 2003). The age-related differences in emotional impression and the age-related differences in behavior were both less pronounced for happy than angry faces.

In summary, for angry faces, associations between subjective emotional impression and behavior were found in two out of three tasks. The lack of an association for happy faces are most likely due to a lower degree of variance in the processing of happy than angry faces.

### Potency as a predictor of emotional behavior

When analyzed as univariate predictors, both high arousal and high potency ratings were associated with an increase in visual search efficiency (i.e., lower RTs). However, after adjustments for arousal and valence in Model 2 and arousal, valance, age in Model 3, only high potency ratings predicted a reduction in RT for angry faces. A likely explanation of this might be the relatively high inter-correlation between the dimensions of arousal and potency (*r* = 0.539). Thus, although arousal may in itself have an effect on visual search performance, as indicated by the results of Model 1, this effect may have been masked by the statistically stronger effect of potency. Hence, although our prediction was of an association between arousal and behavior, our finding that potency is the stronger predictor is neither controversial nor in direct disagreement with our previous results. As mentioned in the introduction, the study of emotional dimensions beyond valence and arousal has been proposed previously (Russell and Mehrabian, 1977; Fontaine et al., 2007). Given that we have found associations between potency and search efficiency in the current study and in a previous one (Lundqvist et al., 2013a)—and an additional association between potency and VSTM performance in the current study—this dimension clearly deserves to be included and investigated in future studies in this field of research.

### Limitations

Using the same stimuli and the same study population across four tasks, we have consistently demonstrated a less accurate processing of angry faces in older than younger adults. Moreover, we found an association between emotional impression and emotional behavior, which indicates that subjective emotional impression influences behavior. However, some limitations are worth mentioning.

For example, the facial stimuli used all depicted younger adults. Although previous research on the recognition of facial expressions has demonstrated equal recognition processing of younger and older faces (Ebner and Johnson, 2009; Ebner et al., 2013), interactions between the age of poser and the age of participants might be present in other domains, such as those used in the present study. Thus, replication of the current study using both younger and older faces would increase the generalizability of our results.

A second potential limitation is the lack of an association between subjective emotional impression and categorical perception performance. A loss of almost 12% of the participants during the categorical perception task might have been sufficiently large to hinder the detection of such an association. The lack of an association might also be linked to nature of the task itself. Whereas the visual search task is related to response speed and the VSTM task is related to updating of information, the categorical perception task taps into categorical judgments or comparisons of two stimuli. Thus, this kind of more elaborative comparison ability might rely on a different behavior component than memory and attention processes, one that is hypothetically less affected by subjective emotional impression.

A third limitation is that because of the exploratory nature of this first study on emotional impression and behavior, we did not correct for multiple comparisons in the current ANOVA or regression analyses. The current results must therefore be considered preliminary until they are replicated in future studies.

However, despite these potential shortcomings, our results uniquely and clearly demonstrate the importance of taking the emotional impression of the stimuli (including all three of the major emotional dimensions) into account in the study of emotional processing in general, and in aging research in particular.

### Adult aging research and processing of emotional stimuli

The results of the current study point to the importance of stimulus selection in studies of emotional behavior in younger and older adults. As shown in the current study, emotional faces elicit different emotional impressions in younger and older adults, and this in turn affects attention and memory processing. The present results are in line both with the memory literature, which shows that people remember material and events of emotional character better than those that are neutral (e.g., see Buchanan, 2007 for a review) and recent attention literature, which has found that arousal affects attention processing (e.g., Lundqvist et al., 2013b). Thus, a decrease in emotional impression might dampen the effect of emotion on memory performance and attention processing. By assuming that younger and older adults perceive emotional material equally in terms of the key dimensions of emotional impression, we risk misattributing age effects in emotional behavior. As our results indicate, a decrease in subjective emotional impression of angry faces was associated with poorer performance on memory and attention processing tasks. An interesting topic for future research would be to more systematically manipulate emotional impression and study subsequent emotional behavior in younger and older adults.

## Conclusions

The results of all of the four tasks used in this study showed an age-related flattening of emotional impression of facial expressions that was more pronounced for angry than for happy faces. The lower level of emotional impression of angry faces in older adults was also mirrored in the two measures of emotional behavior (visual attention and VSTM) for the facial expressions. Regression analyses confirmed the association between emotional impression and behavior by showing that higher potency ratings predicted lower RTs in the visual search task and fewer errors in the VSTM task. These relationships were age-independent but statistically significant only for angry faces. The present findings of age differences in subjective emotional impression should be considered as a possible explanation of age differences in emotional effects on attention and memory in future studies.

### Conflict of interest statement

The authors declare that the research was conducted in the absence of any commercial or financial relationships that could be construed as a potential conflict of interest.
